# Understanding nucleic acid sensing and its therapeutic applications

**DOI:** 10.1038/s12276-023-01118-6

**Published:** 2023-11-09

**Authors:** Ling-Zu Kong, Seok-Min Kim, Chunli Wang, Soo Yun Lee, Se-Chan Oh, Sunyoung Lee, Seona Jo, Tae-Don Kim

**Affiliations:** 1 Immunotherapy Research Center, Korea Research Institute of Bioscience and Biotechnology, Daejeon, 34141 Republic of Korea; 2 Department of Biochemistry, College of Natural Sciences, Chungnam National University, Daejeon, 34134 Republic of Korea; 3 Department of Life Sciences, Korea University, Seoul, 02841 Korea; 4 Department of Functional Genomics, KRIBB School of Bioscience, Korea University of Science and Technology (UST), Daejeon, 34113 Korea; 5 Biomedical Mathematics Group, Institute for Basic Science (IBS), Daejeon, Republic of Korea; 6 Department of Biopharmaceutical Convergence, School of Pharmacy, Sungkyunkwan University, Suwon, Republic of Korea

**Keywords:** Innate immunity, Immunotherapy

## Abstract

Nucleic acid sensing is involved in viral infections, immune response-related diseases, and therapeutics. Based on the composition of nucleic acids, nucleic acid sensors are defined as DNA or RNA sensors. Pathogen-associated nucleic acids are recognized by membrane-bound and intracellular receptors, known as pattern recognition receptors (PRRs), which induce innate immune-mediated antiviral responses. PRR activation is tightly regulated to eliminate infections and prevent abnormal or excessive immune responses. Nucleic acid sensing is an essential mechanism in tumor immunotherapy and gene therapies that target cancer and infectious diseases through genetically engineered immune cells or therapeutic nucleic acids. Nucleic acid sensing supports immune cells in priming desirable immune responses during tumor treatment. Recent studies have shown that nucleic acid sensing affects the efficiency of gene therapy by inhibiting translation. Suppression of innate immunity induced by nucleic acid sensing through small-molecule inhibitors, virus-derived proteins, and chemical modifications offers a potential therapeutic strategy. Herein, we review the mechanisms and regulation of nucleic acid sensing, specifically covering recent advances. Furthermore, we summarize and discuss recent research progress regarding the different effects of nucleic acid sensing on therapeutic efficacy. This study provides insights for the application of nucleic acid sensing in therapy.

## Advancements in tumor immunotherapy: the power of nucleic acid sensing

The study highlights the role of nucleic acid sensors in recognizing and destroying microbes, and their potential as targets for cancer therapy and autoimmune disease treatment. Researchers found that modifying these sensors can improve the efficiency of mRNA vaccines and reduce their immunogenicity. Additionally, understanding the crosstalk between different nucleic acid sensing pathways can help develop new optimization strategies for cancer immunotherapy. These findings pave the way for novel therapeutic approaches that harness the power of the immune system to combat various diseases.

This summary was initially drafted using artificial intelligence, then revised and fact-checked by the author.

## Introduction

As a complementary host defense mechanism, the vertebrate immune system consists of both innate and adaptive immune responses^1^. Although innate immunity cannot confer specificity for host defense or form immune memory, its defense mechanisms can recognize and destroy most microbes within minutes to hours. Cells detect external components of pathogens, or pathogen-associated molecular patterns (PAMPs), through pattern recognition receptors (PRRs), which largely comprise Toll-like receptors (TLRs) and C-type lectin receptors (CLRs). In addition, endosomal TLRs and cytoplasmic nucleic acid receptors, including nucleotide-binding and oligomerization domain NOD-like receptors (NLRs), retinoic acid-inducible gene-I (RIG-I)-like receptors (RLRs), absent in melanoma 2 (AIM2)-like receptors (ALRs), and cyclic GMP-AMP synthase (cGAS), recognize cell-invading exogenous nucleic acids; adaptive immune cells are among those that can detect exogenous pathogens via these receptors.

Nucleic acids, which are the genetic building blocks of all organisms, are potent PAMPs released during viral infection and are discerned as exogenous nucleic acids by specialized PRRs. Based on their different forms of nucleic acids, pathogen-derived double-stranded RNA (dsRNA), single-stranded RNA (ssRNA), and DNA are recognized by TLR3, TLR7/8, and TLR9, respectively, in human endosomes. In contrast, nucleic acid (NA)-sensing mechanisms in the cytoplasm contribute to immunity mainly by recognizing invading RNA by RLRs and invading DNA by cGAS and interferon gamma-inducible 16 (IFI16). Activated NA-sensing PRRs transduce signals to aptamer molecules or directly recruit downstream proteins that mediate cytokine and type I and III interferon (IFN) production by activating nuclear factor (NF)-κB and interferon regulatory factor (IRF) proteins, respectively^2^.

Nucleic acid sensors not only mediate immune defense against pathogens but also detect tumor-derived DNA to trigger antitumor immune responses. Therefore, nucleic acid receptors are potential targets for cancer therapy^3,4^. Organismal development and aging are accompanied by apoptosis, through which nucleic acids are released from cells^5^. Many inflammatory and autoimmune diseases are associated with the dysfunctional or abnormal activation of nucleic acid receptors, which are considered attractive targets for the development of therapeutic agonists or antagonists^6^. To maintain homeostasis and induce optimal immune responses, multiple mechanisms regulate the NA-sensing factors that distinguish between self- and non-self-derived nucleic acids^7,8^. Nucleic acid sensors have exhibited considerable potential in immunotherapy and the treatment of autoimmune diseases; however, the mechanisms underlying their modulatory roles are unclear. For a long time, innate immune-activating molecules were used as adjuvants in vaccines^9^; however, the immunogenicity of mRNA has been found to be the main factor diminishing the efficiency of mRNA vaccines^10^. Therefore, greater attention is being paid to the development of nucleic acid vaccines that do not activate innate immunity and produce more antigenic proteins. In this review, we discuss recent advances in understanding the mechanisms and regulation of NA-sensing and related signaling in different treatments. To highlight the clinical implications of NA-sensing mechanisms, we outline some ways of evading NA-sensing pathways during therapy.

## Nucleic acid sensing in endosomes

TLRs constitute a class of transmembrane innate immune receptors that are evolutionarily conserved and induce immune responses by recognizing distinctive PAMPs. TLRs are single-transmembrane proteins composed of an extracellular N-terminal domain, which recognizes ligands; a transmembrane domain; and a cytoplasmic Toll/interleukin 1 receptor (TIR) domain. Human NA-sensing TLRs include TLR3, TLR7, TLR8, and TLR9, which localize to the intracellular compartment membranes and recognize viral and bacterial cytosolic components, such as nonmethylated CpG DNA and single- and double-stranded RNA.

### Structure and ligands of nucleic acid-sensing TLRs

TLR3, the first described viral TLR, recognizes dsRNAs larger than 40 bp, which are released during RNA virus replication^11^. TLR3-induced responses increase in intensity with increasing dsRNA sequence length; however, the underlying molecular mechanism underlying this increase remains unclear^12^. Two monomeric forms of TLR in solution bind to the dsRNA ligand to form a dimer; the dimerized TLR3 clamps around the dsRNA without any detectable sequence affinity specificity^13^. In contrast to other NA-sensing TLRs, TLR3 is expressed in immune cells as well as some nonimmune cells, such as neurons and keratinocytes^14^, and its widespread expression enables it to play a crucial role in RNA virus infection.

TLR7 and TLR8 specifically recognize ssRNA in endosomes. TLR7 and TLR8 preferentially bind guanosine and uridine, respectively, but contain other ssRNA-binding sites^15^. TLR9 recognizes ssDNA containing unmethylated CpG sequences (commonly found in bacteria and viruses). TLR9 harbors two DNA-binding sites—CpG- and 5′-xCx-binding sites^16^. RNA:DNA hybrids are also recognized by TLR9^17^.

### Trafficking and activation of nucleic acid-sensing TLRs

**Fig. 1:**
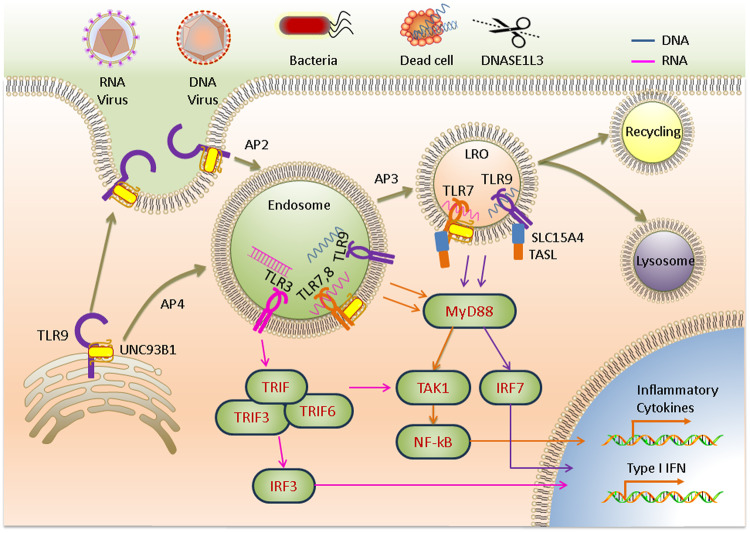
Trafficking of NA-sensing TLRs and downstream signals induced by nucleic acid recognition in endosomes. TLR9 is first trafficked to the plasma membrane and then internalized into the endosome via AP2, where TRIF is recruited to activate downstream transcription factors. TLR7 and TLR9 depend on AP4 for localization to the endosome to activate the TAK1 signaling pathway via the recruitment of MyD88. AP3 further mediates TLR localization to lysosome-related organelles (LROs), where type I IFN gene activation is mediated.

All NA-sensing TLRs are synthesized in the endoplasmic reticulum (ER) and transported to endosomes via the canonical secretory pathway^18^. However, the characteristics of the transport routes and compartments in which they ultimately reside are surprisingly diverse^19^. Unc93B1, an ER multiple transmembrane protein, is an essential trafficking molecule for all NA-sensing TLR proteins^20^ that mediates the differential transport of TLRs^21^. Inactive TLR9 is native to the ER of dendritic cells (DCs) and B cells, from which it is transported first to the cytoplasmic membrane and then internalized into endosomes via adaptor protein 2 (AP2)-mediated endocytosis^22^, whereas TLR7 recruits AP4 directly for subsequent translocation to endosomes^22^ (Fig. 1). TLRs in endosomes undergo proteolytic cleavage, thereby producing functional receptors that interact with nucleic acid ligands^23^.

Activation of all NA-sensing TLRs is restricted to endosomes^19^. This recognition pattern allows cells to recognize and sequester pathogens in the endosomal compartment without risking infection, and the contents are subsequently sorted for degradation or recycling in a small GTPase-dependent manner. Pathogens enter an endosome via endocytosis. After binding to nucleic acids, a TLR forms a complex, either it’s a hetero or homodimer, and the intracellular TIR domains of the dimerized TLRs come into close contact with each other to activate downstream signal transduction. The signaling cascade depends on the types of ligands, interacting TLRs, and downstream bridging molecules. TLR3 homodimers directly recruit TRIF in response to viral dsRNA binding. Other NA-sensing TLRs trigger the NF-κB and/or IRF signaling pathways via MyD88 to induce cytokine and type I IFN production, promoting inflammatory and antiviral responses, respectively^24^. TIR domain-containing adaptor-inducing interferon-β (TRIF), TNF-receptor-associated factor 3 (TRAF3), and TRAF6 form a complex that activates IRF3 signaling to produce type I IFN^21^. However, TLR7 and TLR9 are dependent on AP3-based transport from the endosome to lysosome-related organelles, which is regulated by the peptide transporter protein solute carrier family 15 member 4 (SLC15A4) in the endosomal compartment^25^. TLR7, TLR8, and TLR9 interact with the TLR adaptor TASL in a lysosomal SLC15A4-dependent manner and activate IRF signaling to produce type I IFN^14,26^ (Fig. 1).

Because nucleic acids can be derived from various sources, the regulation of TLR ligand availability is essential to balance the pathogen-sensing and self-recognition abilities of TLRs and modulate inflammatory responses, which primarily involve ligand internalization, nucleic acid digestion or processing, and the cytoplasmic transport of ligands.

### Regulation of nucleic acid-sensing TLRs

Nucleic acid digestion by nucleases regulates ligand availability. Generally, ligand digestion in the endosomal compartment negatively regulates TLR responses, preventing the generation of autoimmune responses and the excessive activation of antiviral innate immune responses. Nucleases that play a regulatory role in the activation of TLRs include ribonuclease (RNase) T2, deoxyribonuclease (DNase) I-like 3 (DNASE1L3), DNase II, phospholipase D3 (PLD3), and PLD4^14^. RNase T2 is widely expressed in a variety of cell types and negatively regulates TLR3 activation by degrading RNA in endosomal compartments; moreover, it is required for the activation of TLR7 and TLR8^27,28^. RNase T2 deficiency or mutations can cause cystic leukoencephalopathy^29^. The endonuclease DNase I-like 3 is expressed in innate immune cells and degrades nucleic acids carried by dead cells before it is internalized^30^ (Fig. 1). DNASE1L3 possesses a unique positively charged and highly hydrophobic C-terminal domain (CTD) that allows it to digest DNA bound to proteins or lipids, which likely contributes to cell transfection difficulties^31^. Functional mutations in the DNASE1L3 gene cause a rare form of pediatric systemic lupus erythematosus (SLE)^32^. DNase II degrades DNA in the endosomal compartment, while loss-of-function mutations in DNASE2 cause type I interferonopathies^33^. PLD3 and PLD4 degrade TLR7 and TLR9 ligands in endolysosomes. Mice deficient in PLD3 and PLD4 suffer from fatal diseases during the early stages of life^34^. Nuclease deficiency can cause large amounts of nucleic acids to enter the cytoplasm during subsequent endosome rupture, activating the cytosolic NA-sensing pathway and causing type I interferonopathies, which can be alleviated by eliminating type I IFN or blocking TLR trafficking^35^. The physiological characteristics of some nucleases that play partial roles or are functionally redundant, such as RNase A and DNase I, require further study^36^.

The amount of ligand internalized by cells is another critical factor affecting ligand availability. It has been shown that the uptake of extracellular immune complexes containing self-nucleic acids is associated with receptor for advanced glycosylation end products (RAGE)^37^. Self-nucleic acids interact with the antimicrobial peptide LL37 or HMGB1 to promote endosomal uptake of nucleic acids and reduce nuclease degradation, which in turn stimulates the activation of NA-sensing TLRs via self-nucleic acids^38,39^. The transport of ligands from the nuclear endosome to the cytoplasm can reduce the concentration of ligands in the endosome. SIDT1 and SIDT2 can promote dsRNA escape from the endosome into the cytoplasm and activate antiviral immune signaling^40,41^.

## RNA sensing in the cytosol

Notably, NA-sensing TLRs are mostly expressed in immune cells. However, epithelial cells and fibroblasts on the mucosal surface, which are exposed to the external environment and are susceptible to infection, can still produce an effective innate immune response to prevent pathogen proliferation^42^. Different cell types employ different nucleic acid recognition mechanisms to combat viral invasion^43^. Various cytoplasmic RNA-sensing mechanisms have been identified.

### Structure and ligands of RLRs

RLRs have been extensively studied as primary cytoplasmic RNA-monitoring mechanisms. RLRs constitute a class of cytoplasmic RNA helicases that detect viral RNA accumulated during infection or replication in a nonsequence-specific manner and elicit antiviral immune responses through the production of type I IFN^44,45^. In contrast to TLRs, RLRs are expressed by most cell types. The RLR family includes RIG-I, melanoma differentiation-associated gene-5 (MDA-5), and laboratory of genetics and physiology 2 (LGP2). All RLRs have conserved structural domains and contain a central DExD/H-box helicase and CTD. RIG-I and MDA5 also carry two N-terminal caspase recruitment domains (CARDs) that are primarily responsible for signal transduction. In the inactivated state of RIG-I, the CARDs interact with the helicase domain to maintain an autoinhibited conformation. Downstream signaling is initiated by exposure to CARDs when RNA binds to the helicase domain and CTD. This conformational change is thought to be triggered by a V-shaped pincer domain consisting of a unique elbow-shaped helical extension of the CTD with the HEL2 helicase domain^46^.

RIG-I recognizes the 5′-ppp structure of an RNA and the blunt base-paired 5′ end. DsRNA are characterized by these ligand structures. Some RNA secondary structures consist of the genetic material of many RNA viruses that are generally not found in healthy host cells. In addition, RIG-I can be induced to produce a weaker signal by RNA without the 5′-PPP structure^47^. Some differences between RLRs have been described^48^. RIG-I recognizes relatively short dsRNAs, while the ligand preferences of MDA5 have not be fully elucidated; however, it is generally believed that MDA5 preferentially binds to long dsRNAs (>1 kb)^49^. The open C-shaped structure of MDA5 confers the ability to assemble filamentous oligomers along long dsRNAs^50^. LGP2 can bind dsRNA; however, it is thought to regulate RLRs because it lacks an NA-sensing signaling function.

### Activation of RLRs

**Fig. 2:**
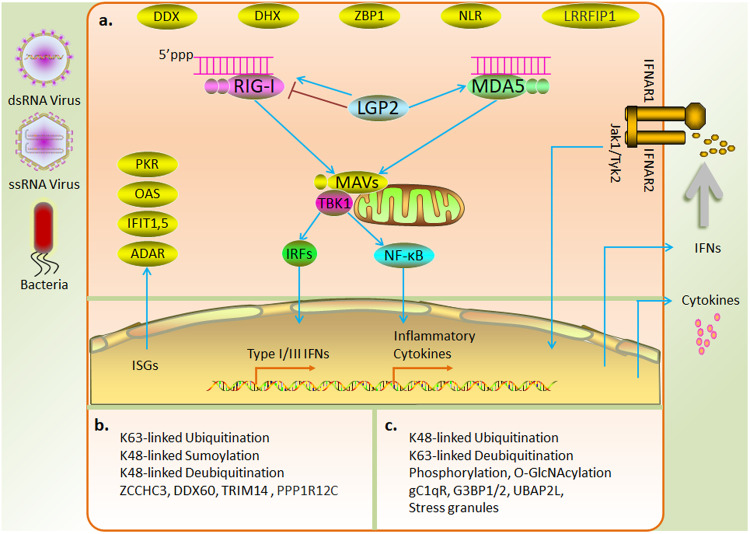
Mechanism and regulation of RNA sensing in the cytosol. **a** RLRs are activated by RNA derived from a virus or bacteria and mediate the production of type I and III IFNs and inflammatory cytokines via the MAVS adaptor protein. Interferons released into the extracellular compartment activate interferon-stimulated genes and induce direct antiviral responses. **b** Positive regulation of RLRs by posttranslational modifications and interacting proteins. **c** Negative regulation of RLRs by posttranslational modifications and interacting proteins.

RLRs exposed to CARDs are fully activated by the action of various enzymes and subsequently depend on interactions with 14-3-3ε^51^ and 14-3-3η^52^, which are members of the 14-3-3 protein family, to mediate the relocalization of RIG-I and MDA5, respectively, to mitochondria. Mitochondrial antiviral-signaling (MAVS) protein is a common adapter protein associated with RIG-I and MDA5 and is localized to the inner mitochondrial membrane. RLRs interact with the homologous CARD of MAVS and subsequently induce TRAF-binding motifs to recruit TRAF2, TRAF5, TRAF6, and TRADD, which mediate the activation of IRF3 or IRF7 via the action of the cytoplasmic kinase TANK-binding kinase 1 (TBK1) to produce type I and III IFNs^15^. In addition, MAVS signaling mediates the stimulation of proinflammatory cytokines through the induction of NF-κB activation via the IKK complex^53^ (Fig. 2a).

LGP2, which lacks a signaling structural domain, has been shown to regulate RIG-I and MDA5 in several studies. LGP2 inhibits RIG-I activation through ligand competition^54^ or by directly impeding the oligomerization and signal activation of RIG-I, which is mediated through the RIG-I CTD domain^55^. In addition, LGP2 interacts with tripartite motif-containing 25 (TRIM25) to inhibit RIG-I ubiquitination^56^. In contrast, LGP2 facilitates MDA5 signaling^57,58^. During viral infection, LGP2 has also shown to promote both RIG1 and MDA5 signaling^59^ (Fig. 2a). In conclusion, the characterization of the regulatory role of LGP2 under specific physiological conditions requires further study.

### Regulation of RLRs

In addition to LGP2, multiple intracellular mechanisms participate in the regulation of RLR activity, including multiple posttranslational modifications (PTMs) and protein interactions. Ubiquitination of RIG-I CARD via K63 linkages, mediated by the ubiquitinated proteins TRIM25, Riplet, TRIM4, and Mex-3 RNA-binding family member C (Mex3c), promotes RIG-I oligomerization and signal transduction^60–63^; in contrast, polyubiquitination via K48 linkages, mediated by ring finger protein 122 (RNF122), RNF125, Casitas B-lineage lymphoma (c-Cbl), and TRIM40^64^, induces RIG-I degradation. Deubiquitinases, including ubiquitin-specific peptidase 3 (USP3), USP21, and CYLD lysine 63 deubiquitinase (CYLD), attenuate the antiviral response by removing the K63-linked polyubiquitin chain. In contrast, USP4 and USP15 enhance the stability of RIG-I by hydrolyzing the K48-linked ubiquitin chain and exerting a positive regulatory effect^65^. SUMOylation prevents RLR degradation via K48-polyubiquitin-dependent degradation, thereby stabilizing RLR in the early stages of viral infection^66^. Additionally, phosphorylation causes RIG-I to be autoinhibited^67^. A recent study showed that O-GlcNAcylation inhibited RIG-1 signaling by modifying MAVS^68^ (Fig. 2b, c). Although the PTMs related to MDA5 signaling have been studied relatively rarely, it is likely that PTMs regulate MDA5 in a manner similar to their regulation of RIG-I.

Many dsRNA-binding proteins participate in the regulation of RLRs. PACT positively regulates RLRs by interacting with the CTD of RIG-I or promoting MDA5 oligomerization^69,70^. The zinc finger protein ZCCHC3 has recently been shown to function as a coreceptor for RIG-I and MDA5^71^. DExD/H-box helicase 60 (DDX60) promotes RIG-I-dependent innate immune responses^72^. In addition to covalent modifications, TRIM14 enhanced RIG-I signaling by recruiting NF-κB essential regulator (NEMO) to the MAVS complex via the ubiquitin chain^73^. A recent study demonstrated that PPP1R12C relocalization triggered by viral infection or RNA delivery reagents promoted downstream signaling by mediating the dephosphorylation of RLRs^74^. In contrast, the complement component C1q (gC1qR) on mitochondria inhibited RIG-I- and MDA5-dependent antiviral responses^75^. Stress granules formed by the aggregation of the key nucleating factors G3BP1/2 and UBAP2L with stalled ribosome–mRNA complexes inhibited excessive activation of RLR signaling and prevented viral replication through unknown physiological functions^76^ (Fig. 2b, c).

### Other RNA sensors

Several other cytoplasmic RNA sensors trigger antiviral responses via transcription factors, including certain DExD/H-box RNA helicases (which recognize RNA through their conserved motifs and are involved in the activation of TLR and RLR downstream signaling pathways), NLRs (which induce inflammasome activation by binding RNA), the LRR domain of flightless-1-interacting protein 1 (LRRFIP1, which binds dsRNA and dsDNA to induce type I IFN production through β-catenin phosphorylation), Z-DNA binding protein (ZBP1^77^, which induces activation of innate immunity and PANoptosis through recognition of Z-DNA and Z-RNA) (Fig. 2a), and HMGB (which may act as a cosensor for various PRRs) (see reviews^47,78^).

Various RNA sensors with direct antiviral activity are expressed in cells; these sensors include 2′,5′-oligoadenylate synthetase (OAS), RNA-regulated protein kinase (PKR), IFN-induced protein with tetratricopeptide repeats 1 (IFIT1), and adenosine deaminase acting on RNA (ADAR), the expression of which depend on type I IFN or PRR signaling^7,79^ (Fig. 2a). OAS binds dsRNA and catalyzes the generation of 2′-5′-linked oligoadenylates (2–5A) from substrate ATP to degrade virus-derived dsRNA by mediating the activation of RNase L. PKR can be activated by viral-derived dsRNA or short 5′-ppp RNA-containing secondary structures. Activated PKR mediates the phosphorylation of the α-subunit of eukaryotic initiation factor 2 (eIF2) to inhibit translation initiation. IFIT1 binds to ssRNAs containing the 5′-ppp terminus to repress cap-dependent RNA translation. ADAR-edited cell-derived self-RNAs can evade NA-sensors; however, A-I editing may lead to amino acid substitutions and loss of function of viral proteins^80^.

## DNA sensing in the cytosol

**Fig. 3:**
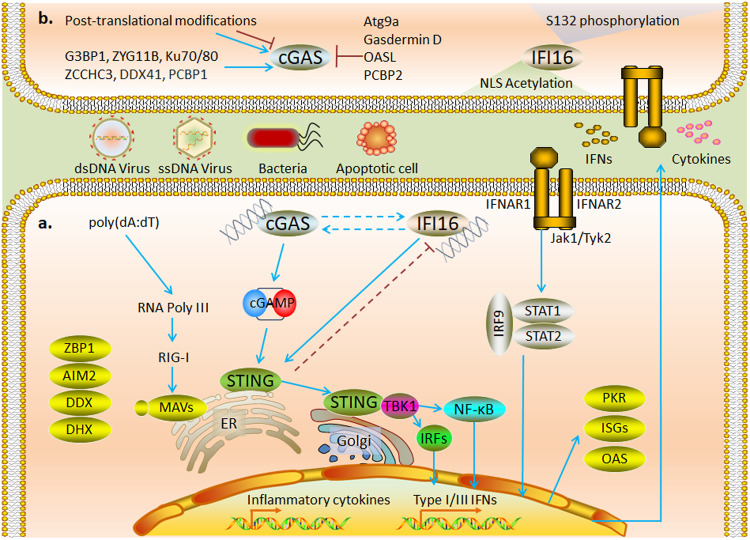
Mechanism and regulation of DNA sensing in the cytosol. **a** cGAS and IFI16, as major DNA receptors in the cytoplasm, induce STING-dependent inflammatory cytokines and IFN production and inhibit viral replication by activating interferon-stimulated genes. **b** Posttranslational modifications of amino acid residues at different sites regulate the activity of cGAS and nuclear localization of IFI16; a variety of proteins have been shown to participate in regulating the activity of cGAS.

Cells infected with a DNA virus but that do not express TLR9 produce high levels of type I IFN^81^. Therefore, ZBP1^82^ and RNA Pol III^83^ were initially identified as cytoplasmic DNA sensors. However, subsequent studies revealed that RNA Pol III-mediated innate immune responses were dependent on poly (dA:dT)-converted RNA ligands with 5′-triphosphate and double-stranded secondary structures to activate the RIG-I/MAVS pathway (Fig. 3a), and interferon production was induced in mouse cells lacking MAVS. Similarly, ZBP1 plays a role only in specific cell types, suggesting that DNA activates unknown DNA-sensing pathways in the cytoplasm in a nonsequence-specific manner.

### Structure and ligands of cytosolic DNA sensors

Interferon-gamma inducible protein 16 (IFI16) and cGAS have been identified as cytoplasmic DNA receptors. Mammalian cGAS belongs to the cGAS/DncV-like nucleotidyltransferase (CD-NTase) family, the members of which are structurally similar to OAS^84^. cGAS contains a disordered N-terminus that anchors its inactivated form to the inner cell membrane^85^, a central NTase domain, and a C-terminal Mab-21 homology domain containing the zinc-ribbon/thumb motif. cGAS binds to dsDNA to form a dimer, followed by DNA sequestration via liquid-phase condensation^86^. cGAS–DNA condensation protects the DNA against Trex1 nuclease-mediated DNA degradation^87^. cGAS activation by dsDNA is DNA length dependent^88^, as more than 45 bp of a dsDNA molecule binding to the A and B sites of each hcGAS molecule and with a third binding site that promotes the stability of the complex.^89^. In addition, cGAS generates innate immunity by recognizing RNA:DNA hybrid molecules generated by intracellular reverse transcription of the HIV-1 virus^90^. PQBP1 acts as an intracellular receptor by which HIV cDNA is recognized by cGAS^91^.

IFI16 (p204 in mice), a member of the ALR family, contains a pyrin structural domain (PYD) and two DNA-binding hematopoietic interferon-inducible nuclear antigens with 200-amino-acid repeat (HIN) structural domains. IFI16 also binds to dsDNA in a length-dependent manner^92^. When binding dsDNA molecules, the PYD structural domain of IFI16 assembles into filamentous oligomers in synergistic association with neighboring PYDs and induces STING-dependent type I IFN production^93^. In addition, IFI16 recognizes viral RNA, promotes RIG-I activation through direct interaction, and upregulates RIG-I transcription by recruiting RNA polymerase II, which provides evidence of crosstalk between RNA- and DNA-sensing mechanisms^94^.

### Activation of cytosolic DNA sensors

Activation of cGAS requires nuclear export signals to mediate its cytoplasmic localization^95^. cGAS catalyzes the generation of the second messenger cGAMP from ATP and GTP, induces IFN production through activation of the STING-TBK1-IRF3 axis, and mediates cytokine production through activation of NF-κB. IFI16 shuttles between the nucleus and cytoplasm and mediates interferon production via a STING-dependent cytosolic signaling pathway^92,96^ (Fig. 3a). In a sequencing analysis of four cell types, IFI16 was found to exert a crucial effect on the transfection efficiency of plasmid DNA (pDNA)^97^.

STING is predominantly located on the ER outer membrane and is expressed in most cells. STING mediates the cytoplasmic dsDNA-induced antiviral innate immune response as an adaptor molecule in response to cGAS and IFI16. cGAMP directly binds to STING, induces STING movement from the ER to the Golgi apparatus, and ultimately recruits TBK1 to colocalize with STING puncta in the perinuclear region. TBK1 recruitment is critical for STING-mediated IRF3 and NF-κB activation^98^. DNA-bound IFI16 interacts with STING in the cytoplasm to recruit and activate TBK1-IRF3 signaling and mediate IFN production^99^ (Fig. 3a).

### Regulation of cytosolic DNA sensors

cGAS is strictly regulated to produce a balanced immune response^100^. Intracellular nucleases are essential for ligand availability in cytoplasmic DNA sensors. Deficiency or mutation in TREX1, RNASEH2, or SAMHD1 leads to cGAS-dependent type I IFN production^101–103^. In addition, multiple mechanisms participate in the regulation of the posttranslational modifications of cGAS^104^. Elimination of the K48-linked ubiquitinated chain suppresses P62-mediated autophagic degradation of cGAS^105^. However, the abrogation of K63-linked polyubiquitination promotes the DNA-binding ability of cGAS^106^. Interestingly, the deubiquitinating enzyme OTUD3 promotes cGAS-mediated DNA sensing but inhibits RLR-mediated RNA sensing^107^. The acetylation of lysine residues in the unstructured N-terminal region of hcGAS promotes its activation^108^. In contrast, acetylation of Lys384/Lys394/Lys414 inhibited cGAS activation^109^. SUMOylation at different sites exerts different regulatory effects on cGAS. SENP2-mediated deSUMOylation induces cGAS degradation during late viral infection^110^. However, SENP7-mediated deSUMOylation enhances cGAS activation^111^. AKT, CDK1, DNA-PK, and Aurora A-mediated phosphorylation of hcGAS can inhibit its enzymatic activity^112–114^. O-GlcNAcylation has been reported to regulate NA-sensing in various cells, although the mechanism remains unclear^115^. OGT has recently been found to activate cGAS-mediated innate immune responses by enhancing the stability of SAMHD1, thereby promoting intracellular dNTP depletion and generating DNA replication intermediates^116^ (Fig. 3b).

High acetylation and phosphorylation rates of endogenous IFI16 have been found in lymphocytes and are mainly associated with nuclear localization. IFI16 carries a nuclear localization signal (NLS) at the N-terminus, and the NLS motif is modified by acetyltransferase p300 to promote accumulation in the cytoplasm^117^. In contrast, phosphorylation by CD2 on S132 promotes the nuclear localization of IFI16^118^. Recent studies revealed that clearly localized IFI16 prevented DNA viral invasion via its effect on different pathways^119^ (Fig. 3b).

Several proteins have been found to mediate cGAS signaling by interacting with ligands or regulating ligand action. Among these proteins, G3BP1, ZYG11B, Ku, and ZCCHC3 promote cGAS-mediated innate immune responses by facilitating DNA binding and condensation^120–123^. DEAD-box helicase 41 (DDX41) promotes cGAS activation by regulating DNA stabilization via its helicase activity^124^. Others, such as Atg9a and Gasdermin D, inhibit STING-dependent innate immune responses by mediating autophagy^125,126^. OASL suppresses IFN production by specifically binding to cGAS during DNA virus infection^127^. Notably, poly(rC)-binding protein 1 (PCBP1) facilitates the binding of cGAS to DNA, whereas PCBP2 interacts with cGAS and prevents its excessive activation^128,129^ (Fig. 3b). Additionally, cGAS, IFI16, and STING regulate each other. cGAS may contribute to the innate immune response by increasing the stability of IFI16^130^, and IFI16-mediated TBK1 recruitment is essential for cGAMP-mediated STING activation^96,131^. STING negatively regulates antiviral immune responses through TRIM21-mediated ubiquitinated degradation of IFI16^132^. Moreover, the transport of extracellular second messenger cyclic dinucleotides (CDNs) by SLC19A1^133^, SLC19A2^134^, LRRC8^135^, LL37^136^, P2X7R^137^, and Connexin^138^ is essential for the activation of intracellular STING. ABCC1 has recently been identified as a cGAMP export protein^139^. ENPP1 attenuates STING activation in bystander cells by degrading extracellular cGAMP^140^.

### Other cytosolic DNA sensors

Other DNA sensors can recognize DNA in specific cell types or may recognize only specific sequences, mainly, the DExD/H-box helicases DHX9 and DHX36 (which recognize CpG DNA to activate the TLR downstream signaling pathway), DDX41 and DDX60 (which enhance the type I IFN response by binding dsDNA), and AIM2 (which triggers the inflammasome pathway by binding dsDNA to produce IL-1β and IL-18) (see reviews^42,141^) (Fig. 3a).

## Nucleic acid sensing as a promising therapeutic target

NA-sensing exerts both pro- and antitumor effects at different stages of tumorigenesis. Genomic instability typically produces autoimmunogenic DNA in cancer cells. Therefore, NA-sensing-mediated IFN production contributes to DC maturation and tumor-specific T-cell responses^142^. However, in vitro studies have revealed that NA-sensing pathways in several cancer cells are inhibited by JAK2-STAT3-mediated signaling^143^. External activation of NA-sensing has shown enhanced antitumor effects in a variety of cancers. However, in metastatic cancer, the cGAS-STING-TBK1 axis-mediated inflammatory response is positively associated with tumor metastasis. These opposing effects may be associated with the type and stage of the tumor^8^.

NA-sensing-associated mechanisms also play different regulatory roles in gene therapy. Genetic vaccines, including DNA and RNA vaccines consisting the nucleic acids of target genes, are injected directly into the body to induce innate and adaptive immune responses^144,145^. pDNA is an intrinsic adjuvant for DNA vaccines and is essential for the activation of resident antigen-presenting cells through activation of the innate immune response via the action of STING-TBK1^146,147^. However, type I IFN produced by activated nucleic acid induction inhibits the translation of mRNA vaccine-encoded antigenic proteins, thereby reducing antigen-specific immunity^148,149^. Type I IFN is probably critical for enhancing the early immune response but is also the main cause of side effects^150^. For optimal treatment outcomes, it is essential that the immunostimulation and transfection efficiency of nucleic acids be balanced when designing therapeutic strategies^151^.

### Positive regulation of nucleic acid sensing in therapy

**Fig. 4:**
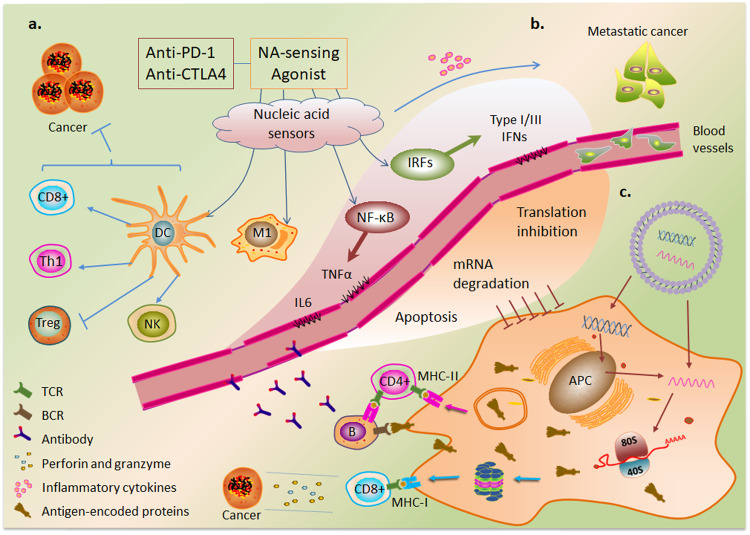
Differential regulation of NA-sensing signaling pathways in therapy. **a** NA-sensing promotes the antitumor therapeutic efficacy of immune checkpoint inhibitors by inducing dendritic cell (DC) maturation and tumor-specific T-cell responses and promotes the differentiation of macrophages into M1 proinflammatory macrophages. **b** In the metastatic stage of cancer, NA-sensing-induced inflammatory cytokines exhibit cancer-promoting effects. **c** Model of antigen-specific immunity mediated by nonviral gene therapies and the negative regulatory effects of NA-sensing on therapeutic transgenes.

The activation of NA-sensing in cancer cells promotes hot tumor transformation through the production of type I IFN and cytokines^152^. Type I interferons also upregulate the expression of major histocompatibility complex (MHC) class I molecules in antigen-presenting cells, which present processed cancer cell-derived antigen molecules to CD8+ T cells^152^. Stimulating the production of type I and III IFNs in CD4+ T cells confers self-protection against HIV infection and enhances the ability of CAR-T cells to clear tumor cells^153^. In addition, cGAS-mediated cGAMP release from cancer cells activates adjacent immune cells^154,155^. Studies have shown that the DNA released from tumor cells after chemotherapy and radiotherapy activates NA-sensing signals that synergistically enhance antitumor effects^156–158^. Agonists of cGAS, STING, and RIG-I potentiate the antitumor activity of immune cells^159–161^; for example, the combination of a STING agonist and a PD1 blocker showed therapeutic effects in tumors with low immunogenicity^162^. Similarly, the innate immune response mediated by RIG-I ligands in combination with CTLA-4 blockade enhanced adaptive immune response-mediated antitumor effects^163^. Furthermore, this combination therapy can enhance the antitumor effect of the anti-PD1 antibody in a cGAS-dependent manner by inhibiting the protein arginine methyltransferase PRMT1- and PRMT5-mediated methylation of the cGAS residues Arg133 and Arg124, respectively^164,165^ (Fig. 4a). A recent study suggested that inducing RIG-I-dependent OAS/RNase L-mediated apoptosis is a potential strategy for cancer immunotherapy^166^.

Because the basic components of pDNA, such as the TLR9 agonist, are immunogenic, the unmethylated CpG sequence is commonly used as a vaccine adjuvant^167^. CpG oligodeoxynucleotides (ODNs) stimulate the maturation and survival of plasmacytoid DCs and accelerate regulatory T (Treg) cell differentiation and depletion through the activation of TLR9^168,169^. Recent studies revealed that SARS CoV-2 mRNA vaccination exposes HIV to CD8+ T cells^170^. A small-molecule agonist of RIG-I, KIN1148, exhibits an adjuvant effect on influenza virus vaccine immunity^171^. The dsRNA analog poly(I:C) activates TLR3 and MDA5 to induce Th1 cell and CD8+ T-cell immune responses through the production of IFN and cytokines^172^. Activation of TLR7/8 and the RIG-I pathway promotes macrophage differentiation toward the M1 proinflammatory phenotype and exhibits antitumor activity^173,174^ (Fig. 4a). In summary, NA-sensing has emerged as a promising target for cancer immunotherapy^175^.

### Negative regulation of nucleic acid sensing in therapy

The disadvantages of intrinsic NA-sensing activation are mainly observed in autoimmune and inflammatory diseases^176^. NA-sensing is especially important during the metastatic stage of cancer and is activated under specific conditions. Increased levels of inflammatory factors caused by NA-sensing have been associated with poor prognosis^8^ (Fig. 4b). Recent studies have shown that RIG-I attenuates the tumor-killing effect of CD8+ T cells by inhibiting STAT5 action^177^.

Although the innate immune response induced via nucleic acid immunity can contribute to disease attenuation, it also plays a negative regulatory role. NA-sensing induces apoptosis in host cells via multiple pathways^178^. IRF3-mediated apoptosis impairs T-cell proliferation and metabolism^179^. Mechanistically, activated IRF3 binds to the proapoptotic protein Bax, and the subsequent translocation of the IRF3-Bax complex to mitochondria promotes the release of cytochrome c into the cytoplasm, thereby inducing apoptosis^8,180^. In contrast, NA-sensing mediates the degradation of transfected RNA and inhibits translation initiation through the actions of interferons^181^. OAS recognizes dsRNA and activates RNase L to mediate RNA degradation. The degraded RNA can also activate other NA-sensing PRRs^182^. dsRNA-dependent activation of PKR subsequently phosphorylates translation initiation factor eIF2α, resulting in translation repression^183^. IFIT1 can also suppress translation by sequestering eukaryotic initiation factors or directly binding to the 5′ end of foreign RNA^184^ (Fig. 4c).

mRNA vaccines elicit different immune responses by encoding antigenic proteins. On the one hand, mRNA-encoded proteins acting as endogenous antigens are degraded by proteasomes into antigenic peptides and activate CD8+ T cells via MHC class I molecules. On the other hand, mRNA vaccine-encoded proteins secreted into extracellular compartments are internalized by antigen-presenting cells, which generate antigenic peptides by proteolysis in endosomes and are presented to CD4+ T cells via MHC class II molecules, which can induce cytokine secretion and stimulate B cells to activate humoral immune responses^185^ (Fig. 4c). The induction of these immune responses depends on the transfection efficiency of the mRNA vaccine and is inhibited mainly by negative regulatory effects mediated by NA-sensing^149^. NA-sensing also causes gene editing difficulty in some cells. Inhibition or evasion of NA-sensing can save nucleic acids from translational repression, thereby improving gene transfection efficiency and increasing the expression of functional protein products^186,187^.

### Strategies to evade nucleic acid sensing

#### Small-molecule inhibitors and viral proteins

Small-molecule inhibitors (see review^188^) of DNA sensing pathways have potential therapeutic value in diseases with long-term activation of proinflammatory pathways, such as autoimmune and inflammatory diseases^161,189,190^. In addition, A151 ODN inhibits the activity of multiple DNA receptors^191^, and 2′-O-methyl (2′OMe) gapmer-modified antisense oligonucleotides show sequence-dependent inhibition of NA-sensing mediated via RNase-H1 recruitment^192^.

Understanding how viruses evade immune recognition is important for antiviral research and immunotherapy^193^. Multiple virus-encoded proteins inhibit NA-sensing-associated pathways (see review^194^). Vaccinia virus (VACV), the most studied Poxviridae^195^, degrades cGAMP via *B2R* gene-encoded POXIN^196^. Some viruses are thought to improve the efficiency of nucleic acid vaccines by blocking the RNA-sensing pathway and enhancing gene expression^197^. Among these proteins, influenza A virus nonstructural protein 1 (NS1) stimulates mRNA translation by inhibiting interferon production^198^. Vaccinia protein B18R inhibits type I IFN to enhance mRNA stability and translation efficiency^199^.

#### Sequence optimization and chemical modifications

Nucleic acid modification can prevent the innate immune response-mediated translational repression of exogenous genes by reducing immunogenicity^186^. The 5′-cap1 structure (a 2′-O-methyl group linked to the first nucleotide: m7GpppNmpN) can escape RIG-I recognition, thereby increasing translation efficiency^200,201^. The addition of poly(A) tails minimizes mRNA immunogenicity by reducing the U content of the sequence^186^. Circular RNAs (circRNAs) reportedly exhibit low immunogenicity and high stability and can initiate stable translation via internal ribosome entry site elements^202,203^. The incorporation of N6-methyladenosine (m6A)-modified circRNAs completely abrogated RIG-I-mediated activation of the immune response^204^. In addition, many chemical modifications of RNA bases have been leveraged to reduce the immunogenicity of mRNA; these modifications include pseudouridine (Ψ), N1-methyl-pseudouridine (m1Ψ), 2-thiouridine (s2U), 5-methoxyuridine (m5U), and 5-methylcytidine (m5C)^205–207^. DNA transfection was performed to construct CAR-modified immune cells, and the low efficiency of pDNA transfection in immune cells was appropriately resolved by removing CpG sequences and reducing plasmid size^208^.

### Limitations and prospects of nucleic acid sensing in therapy

Despite multiple modifications aimed at limiting undesired immune stimulation caused by nucleic acid vaccines, further optimization is necessary to achieve the desired transfection efficiency and economic viability. For instance, although DNA vaccines can trigger an immune response in animal experiments, they exhibit low immunogenicity in human clinical trials^147^, thereby slowing the development of DNA vaccines. DNA vaccines have also been used in the fight against COVID-19; for example, the COVIDITY DNA vaccine was developed with two plasmids encoding the S protein receptor-binding domain and the nucleocapsid (N) protein, thus providing a mechanism to enhance extracellular antigen cross-presentation. Nevertheless, although physical delivery methods such as electroporation or needle-free injection systems may address delivery efficiency issues, the risk of DNA insertion remains a concern^209^. In contrast, agonists and antagonists of DNA sensors show more promise for clinical applications^6^. In contrast to DNA vaccines, RNA vaccines carry no risk of genomic insertion and are easy to deliver. Although balancing antigen expression and immunogenicity of RNA can increase the antigen availability, the thermal instability of these vaccines remains a challenge that has not been adequately addressed.

As mentioned earlier, NA-sensing activation exhibits both benefits and drawbacks in disease treatment, and its necessity must be carefully evaluated in the context of different diseases and stages of pathogenesis. The study and comparison of DNA-sensing and RNA-sensing interactions can help in identifying new optimization strategies^210,211^. The low immunogenicity of DNA vaccines may be due to some degree of cell-type specificity of DNA sensors, but it is unclear where nucleic acid vaccines that are injected into the skin accumulate. Targeted delivery of nucleic acid vaccines to lymph nodes or tumors may reduce NA-sensing while enhancing antigen-specific immune responses^212^. Furthermore, the effect of STING on tumor-associated macrophage differentiation helps alleviate tumor cell-mediated immunosuppression in the tumor microenvironment^213^. An alternative method for engineering T cells is in vivo RNA transfection^214^, although the role of NA-sensing of in vitro transcribed mRNA after CAR transfection remains unclear. A vast body of research links NA-sensing modulation to other therapeutic approaches^215^.

## Conclusions

NA-sensing plays an important role in immunotherapy owing to its ability to elicit innate immunity. Therefore, a comprehensive understanding of the regulation and mechanisms underlying NA-sensing may contribute to the development of antitumor therapies. Several emerging regulatory mechanisms complement the profiling of NA-sensing systems. Although human nucleic acid receptors are diverse, their recognition ligands overlap, and there are similarities in their regulatory mechanisms and downstream signals, such as common adaptor proteins and cofactors. To effectively prevent pathogenic infections, humans have evolved redundant NA-sensing systems to complement the cellular recognition of immunogenic nucleic acids. Therefore, crosstalk among nucleic acid receptors is essential^216^. In this review, we describe the regulatory mechanisms of nucleic acid receptors.

NA-sensing is a double-edged sword in the field of therapeutics. In cancer therapy, NA-sensing tends to have a facilitative effect on antitumor immunity and is thus considered a potential treatment target. However, in the field of gene therapy, it is important to prevent the excessive activation of NA-sensing pathways to maintain proper immunogenicity and efficient gene transfection. The application of in vitro-transcribed mRNA has emerged as a promising therapeutic strategy. Multiple modification approaches have been proposed for increasing therapeutic efficiency by increasing transfection efficiency. In conclusion, nucleic acid sensors are potential targets for gene and cell therapies, which must be generated to balance therapeutic transgene-mediated innate and adaptive immune responses^145,217,218^.
